# A New, Effective and Low-Cost Three-Dimensional Approach for the Estimation of Upper-Limb Volume

**DOI:** 10.3390/s150612342

**Published:** 2015-05-26

**Authors:** Roberto Buffa, Elena Mereu, Paolo Lussu, Valeria Succa, Tonino Pisanu, Franco Buffa, Elisabetta Marini

**Affiliations:** 1 Department of Life and Environmental Sciences, University of Cagliari, Cagliari 09126, Italy; E-Mails: rbuffa@unica.it (R.B.); emereu@unica.it (E.M.); paolo.lussu@gmail.com (P.L.); valerias@unica.it (V.S.); 2 Metrology Group of Sardinia Radio Telescope, INAF, Selargius 09047, Italy; E-Mails: tpisanu@oa-cagliari.inaf.it (T.P.); fbuffa@oa-cagliari.inaf.it (F.B.)

**Keywords:** accuracy and reliability, upper limb volume, three-dimensional technique, lymphedema

## Abstract

The aim of this research was to validate a new procedure (SkanLab) for the three-dimensional estimation of total arm volume. SkanLab is based on a single structured-light Kinect sensor (Microsoft, *Redmond, WA, USA*) and on Skanect (Occipital, San Francisco, CA, USA) and MeshLab (Visual Computing Lab, Pisa, Italy) software. The volume of twelve plastic cylinders was measured using geometry, as the reference, water displacement and SkanLab techniques (two raters and repetitions). The right total arm volume of thirty adults was measured by water displacement (reference) and SkanLab (two raters and repetitions). The bias and limits of agreement (LOA) between techniques were determined using the Bland–Altman method. Intra- and inter-rater reliability was assessed using the intraclass correlation coefficient (ICC) and the standard error of measurement. The bias of SkanLab in measuring the cylinders volume was −21.9 mL (−5.7%) (LOA: −62.0 to 18.2 mL; −18.1% to 6.7%) and in measuring the volume of arms’ was −9.9 mL (−0.6%) (LOA: −49.6 to 29.8 mL; −2.6% to 1.4%). SkanLab’s intra- and inter-rater reliabilities were very high (ICC >0.99). In conclusion, SkanLab is a fast, safe and low-cost method for assessing total arm volume, with high levels of accuracy and reliability. SkanLab represents a promising tool in clinical applications.

## 1. Introduction

The measurement of limb volume is widely employed for diagnosing and monitoring various clinical conditions. A common application is the diagnosis of breast cancer-related lymphedema, a condition occurring in about 20% of breast cancer survivors [1], that can seriously affect quality of life [2,3]. Other fields of interest concern prosthetics [4], biomechanics [5], sport physiology [6] and nutrition (regional body composition analysis [7]).

The widely accepted “gold standard” for the estimation of limb volume in a laboratory context is the water displacement technique, based on the measurement of the amount of water displaced by the limb when immersed in a tank of water [8]. Although generally considered reliable and accurate, this method has inherent technical difficulties: it is time consuming; it requires active cooperation by the subject; and it is contraindicated in the case of wounds, abrasions or burns [8,9].

A commonly-used technique is the circumferential method [9]. Girth measurements are recorded at different levels of the limb, and the volume of each limb section, assumed to be shaped as a truncated cone or a cylinder, is calculated by means of geometric formulas. This anthropometric approach is inexpensive, easy to use and well correlated with water displacement [9,10,11]. Nevertheless, it is prone to errors linked to the poorly standardized procedure and to the technical error of measurement for anthropometry [12]. Another source of error derives from the morphological variability of the limb that cannot be accounted for by predictive formulas assuming regular shapes [13,14,15].

More recently, three-dimensional (3D) imaging techniques, such as laser scanning [16], the projection of structured light patterns [17] and infrared optoelectronic volumetry (Perometer [18]), have been proposed as highly reliable methods. The Perometer was initially validated against the circumferential method [15] and then using water displacement or DXA as references [18,19]. It uses a series of infrared light sources and sensors, in pairs, interfaced with software for the estimation of transversal limb sections and volume. The procedure is fast and safe, can be applied to patients with skin lesions [19] and has been suggested as a reference method [20]. However, the relatively high cost limits its application in medicine [9,21].

Low-cost range sensors represent a promising alternative. Firstly developed for virtual reality videogames, the Kinect sensor (Microsoft, Redmond, WA, USA) uses the projection of a structured light pattern for 3D data capture. It is able to track the position and orientation of a human body and can be used to estimate both whole and segmental body volume. Recent tests using this sensor have been conducted in order to measure anthropometric dimensions and body movement for use in various biomedical fields, such as ergonomics [22] and kinematics [23,24]. However, to the best of our knowledge, there are only a few studies focusing on the use of Kinect for the assessment of total [25] or segmental body volume [26,27].

The aim of this paper was to validate SkanLab, a new procedure designed on the principles of Kinect measurements for the estimation of upper extremity volume. With respect to the alternative Kinect approach for assessing arm volume [27], SkanLab is based on a simpler and lower cost procedure. SkanLab is characterized by high spatial resolution, free available software, portability and non-invasiveness, hence representing a promising tool for clinical application.

## 2. Materials and Methods

The experimental protocol was designed to assess the accuracy and reliability of the newly proposed method by comparison with volumetric reference techniques (Table 1). Volumetric analyses were carried out on twelve inanimate objects (plastic cylinders) and a larger sample of human arms (30 subjects).

**Table 1:** Methods and reference technique applied for volumetric estimations.

Samples	Methods	No. of Raters	No. of Replications by Each Rater	No. of Measurements	Reference Technique
Cylinders (N = 12)	Geometry SkanLab	1	1	12	-
		2	2	48	Geometry
Human Total Arms (N = 30)	Water Displacement SkanLab	2	2	120	-
		2	2	120	Water Displacement

### 2.1. Samples

#### 2.1.1. Inanimate Objects

Three plastic (polyvinyl chloride, PVC) cylinders (diameters: 40, 80 and 110 mm) were marked with plastic tape at different heights, resulting in twelve regularly-shaped objects with different volumes (25.5 mL to 2002.4 mL).

The volume of the cylinders was geometrically determined using structured-light scanning as a reference method, with one rater and one replication for each object. The experimental model for measuring objects with SkanLab was two raters and two replications. For comparative purposes, object volumes were also measured with water displacement (two raters and two replications).

#### 2.1.2. Human Total Arms

In *vivo* experimentation was performed considering a sample of thirty subjects (fifteen men and fifteen women, aged from 19 to 60 years), recruited from the university staff and students by convenience sampling. The study was approved by the ethical committee of Cagliari University Hospital (protocol number: PG/2014/21461). The sample size was comparable to that of similar validation studies [8,9,13,16,28]. In accordance with the Helsinki Declaration of 1964, as revised in 2013, all volunteers were informed about the research protocol, and they consented to take part in the research. Exclusion criteria included: presence of upper extremity lesions, history of cardiovascular or metabolic diseases, cancer, inflammatory conditions and pregnancy.

Anthropometric measurements (stature; body weight; acromion-olecranon distance, indicating total arm length) were taken by an experienced observer according to standard procedures [29].

Volumes were determined using SkanLab and water displacement as reference (two raters and two replications), because the scanner used to measure cylinders was not suitable for volunteers.

### 2.2. Techniques

#### 2.2.1. Geometric Volume Determination

The volume of the cylinders was determined by a skilled operator using a structured-light scanner (HDI Advance R2-3D3 Solutions, Canada) with an accuracy of 65 µm and a precision of volume estimate equal to 0.001 cm^3^.

#### 2.2.2. Water Displacement Method

A volumeter was assembled following indications detailed by Lette *et al.* [30]. The PVC water tank measured 90 × 14 cm, containing approximately 11 L water. Measurements were taken by filling the tank with deionized water to the level of the spout. Water temperature ranged between 20 °C and 25 °C. Celsius at the time of each measurement. The cylinders to be measured were slowly immersed up to the level marked with plastic tape and the displaced water, overflowing through the spout, collected into a beaker. The collected water was then weighed using a laboratory digital scale (ML Systems, Italy; accuracy: 0.1 g) and the volume calculated from water density, after adjusting for temperature.

An iron cylinder (height = 17.0 cm; diameter = 5.1 cm; volume = 351.2 mL) was used for verifying the accuracy and precision of the volumeter. Its mean volume, obtained by ten replicates with the volumeter, was 351.0 mL (CV% = 0.228).

The volume of human total arms was calculated as the difference of total arm and hand volume, the latter being excluded because of its variable and irregular shape [31]. Using a dermographic pencil, the arm of each volunteer was marked normally to the arm axis at the level of the wrist crease distal to the styloid process (minimum wrist circumference) and up to 60% of the distance between the acromion of the shoulder and the olecranon of the ulna. Each volunteer placed their hand in the water up to the wrist mark and then to the arm mark. In each step, the displaced liquid was weighed. Prior to immersion, talcum powder was spread over the arm in order to promote a better view of the water level. A line was then drawn in correspondence to the immersion point.

Each rater registered the duration of the measurement in seconds.

#### 2.2.3. The Newly Proposed Procedure

The procedure assesses the total arm volume with the projection of structured light patterns on the sample surface and the subsequent image acquisition through electronic cameras.

**Figure 1:**
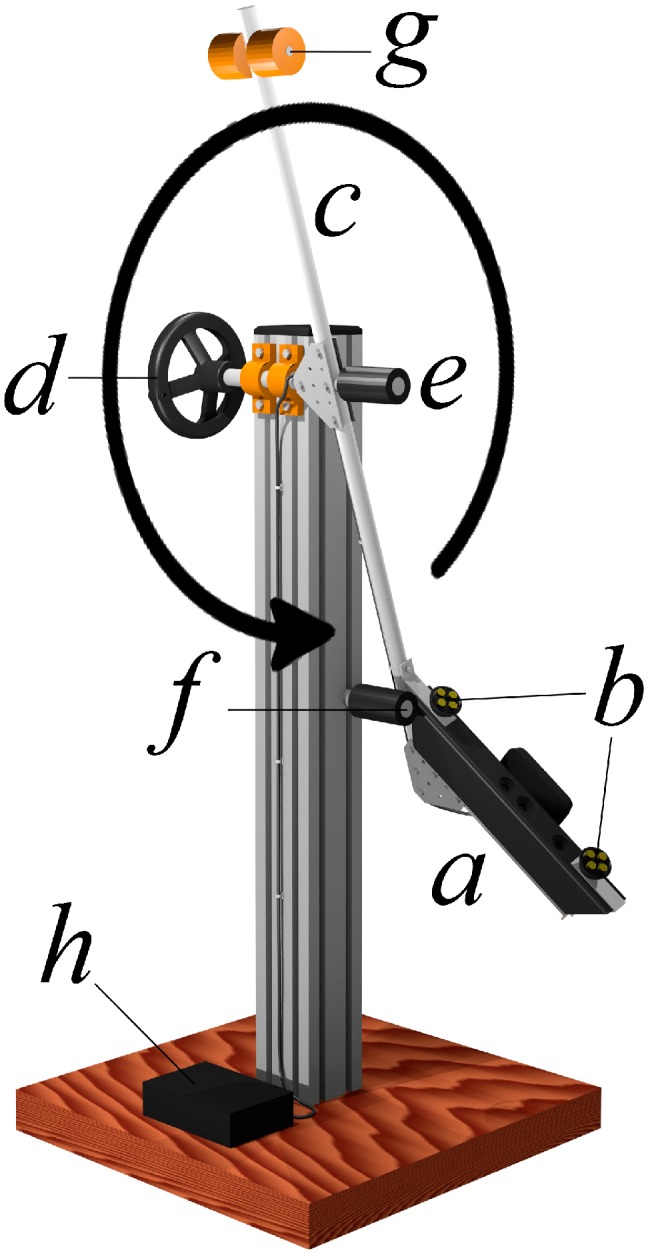
The equipment developed for SkanLab. (**a**) Sensor unit; (**b**) visible light LED sources; (**c**) rotating detection frame; (**d**) manual drive wheel; (**e**) handle; (**f**) frame stop; (**g**) rod and balance weights; (**h**) mains and computer interface.

The hardware includes a stand and a rotating detection frame, both made of aluminum, the latter bearing two visible light LED sources and the Kinect sensor (Figure 1). The length and inclination of the instrument have been specifically designed for scanning the human arm.

The Kinect sensor was chosen for being a low-cost, mass-produced and readily available device, with similarly available application programming interfaces. The sensor comprises a source of structured radiation in the near-infrared spectrum and two electronic CMOS cameras: the RGB camera, which detects the visible light reflected by the points of the sample surface to represent its appearance (color and brightness), and the depth camera, which detects the reflected infrared radiation to estimate the distance. The images produced by the two cameras are processed with 640 × 480 pixel resolution and integrated pixel by pixel in a 3D point cloud. Here, each point has the appearance given by the RGB camera and the distance from the sensor measured through the depth camera. The discriminatory capability of the depth camera is best between 0.6 and 1.8 m; beyond 1.8 m, it decreases while increasing the distance of the sample surface.

The sensor unit slowly rotates around the sample by means of two high precision bearings, capturing new point clouds at a frequency of thirty per second. These are collimated with the previous ones until the entire outer surface of the sample is closed. The LED light sources installed on the rotating arm ensure even illumination of the sample regardless of the angle of view, avoiding changes in appearance due to variations in ambient lighting conditions. In fact, as shown by our preliminary tests and the results of other studies [32], light conditions influence the measurement quality, with the worse performance (missing scan points) under sunlight.

**Figure 2:**
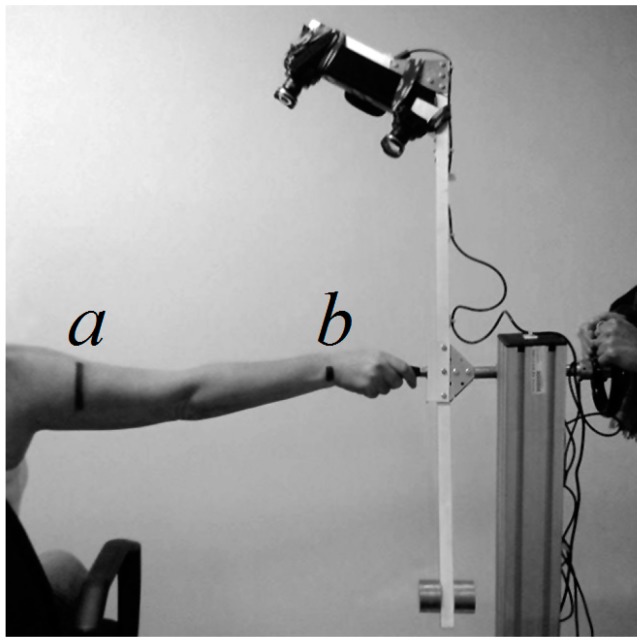
Measurement phase with SkanLab. (**a**) Level marking the 60% of the distance between the acromion of the shoulder and the olecranon of the ulna; (**b**) level marking the wrist crease distal to the styloid process (minimum wrist circumference).

At the beginning of the measurement phase, the sensor unit is placed in its starting position. The subject sits on an adjustable stool, stretches his or her right arm and places his or her hand on the handle in order to keep the arm parallel to the rotation axis and to minimize involuntary movements (Figure 2).

In order to acquire the sample surface as a mesh, a free for non-commercial use software (Skanect 3D Scanning Software by Occipital, free Version 1.7, 2015) was interfaced with the Kinect sensor. This software is able to compensate for minor movements of the subject, and it was used to fill any small gaps in the mesh. Furthermore, it was used to superimpose the information on the position of the points that related to brightness and color. Subsequently, the free and open source MeshLab software, developed by the Visual Computing Lab, “Istituto di Scienza e Tecnologie dell’Informazione A. Faedo” of the National Research Council (ISTI–CNR) of Italy (Pisa, Italy) (Latest Version, V1.3.3, 2 April 2014) [33], was used to remove artefacts from the acquisition process, align all meshes with respect to a common Cartesian reference system, trim off the region of interest and measure its volume.

### 2.3. Statistical Analysis

Statistical analysis was performed to estimate the accuracy and reliability of the new procedure by comparison with geometry and water displacement (for inanimate objects and human arms, respectively).

#### 2.3.1. Accuracy

In accordance with Bland and Altman [34,35], plots were drawn to analyze the consistency between the volumetric methods. Here, bias was defined as the mean difference between measurements obtained with the two techniques on the same object. The 95% limits of agreement (LOA = mean difference ± 1.96 standard deviation, SD) were calculated considering the effect of the replicated measurements. A plot for each comparison (inanimate objects: SkanLab *vs*. geometry; human total arms: SkanLab *vs*. water displacement) was derived.

A positive bias indicates that SkanLab overestimates the value with respect to the reference.

Further, in order to analyze a possible dimensional effect on accuracy, linear regression analysis was applied. The differences between volumes obtained with the reference technique and SkanLab were regressed on cylinder or total arm volume, evaluated by geometry or water displacement, and on BMI.

#### 2.3.2. Reliability

In accordance with Shrout and Fleiss [36], the intra-observer reliability for each rater and technique was estimated by the intraclass correlation coefficient (ICC), using the (2,1) model. The Currier [37] criteria were used to evaluate the ICC results: 0.90–0.99, high reliability; 0.80–0.89, good reliability; 0.70–0.79, fair reliability; less than 0.69, poor reliability. The standard error of the measurement (SEM) was calculated as: SD√(1-ICC).

Inter-observer reliability was measured by the ICC (2,2) model [36], considering the mean of the two measurements taken by each rater on each object. Inter-observer SEM was calculated using the average of the replicated measurements for each rater.

#### 2.3.3. Duration

A Student’s *t*-test was applied to compare the mean duration of arm measurements for both water displacement and SkanLab techniques.

## 3. Results

### 3.1. Inanimate Objects

#### 3.1.1. Accuracy

**Figure 3:**
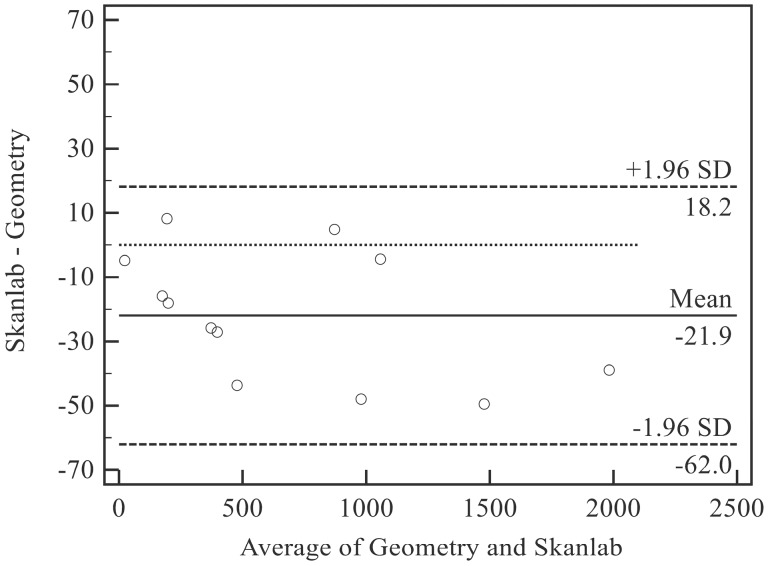
Bias and limits of agreement between SkanLab and Geometry for inanimate objects. SD, standard deviation.

The bias of SkanLab was −21.9 mL (−5.7%) (LOA: −62.0 to 18.2 mL; −18.1% to 6.7%) (Figure 3), with slightly lower mean volumes with respect to those measured by geometry. The two raters showed similar results, with biases ranging between 21.7 mL and 22.2 mL (Table 2, Figure 3).

The linear regression equation (*y* = −0.01 × (−6.02); *R*^2^ = 0.161) showed no significant relationships between the volume measured by geometry (*x*) and accuracy (*y*: volumes measured by geometry minus volumes measured by SkanLab).

#### 3.1.2. Reliability

SkanLab showed very high levels of intra- and inter-rater reliability (Table 3). In fact, the intra- and inter-rater ICC values were near one, falling within the limits of high reliability [37]. The intra- and inter-rater SEM ranged between 5.82 mL and 5.84 mL.

**Table 2:** Accuracy of SkanLab in measuring inanimate objects (cylinders; N = 12).

	Rater 1 Absolute Relative		Rater 2 Absolute Relative	
Bias ^a^	−21.7 mL	−5.7%	−22.2 mL	−5.7%
LOA ^b^	−63.9 to 20.6 mL	−17.9 to 6.6%	−61.7 to 17.4 mL	−19.4 to 8.0%

^a^ Mean difference between SkanLab and geometry and the other techniques (water displacement and SkanLab); ^b^ limits of agreement.

**Table 3:** Reliability of SkanLab in measuring inanimate objects (cylinders; N = 12).

	Rater 1	Rater 2
	Mean ± SD	Mean ± SD
Replicate 1	672.7 ± 597.0 mL	671.5 ± 594.7 mL
Replicate 2	672.7 ± 593.1 mL	674.9 ± 599.6 mL
Intra-rater SEM ^a^	5.82 mL	5.84 mL
Intra-rater ICC ^b^	0.9999 (0.9997 to 1)	0.9999 (0.9997 to 1)
Inter-rater SEM ^a^	5.83 mL	
Inter-rater ICC ^b^	0.9999 (0.9999 to 1)	

^a^ Standard error of measurement; ^b^ intraclass correlation coefficient.

Mean and standard deviation values refer to the measurements taken on the 12 cylinders described in Section 2.1.1. These parameters are intended to compare the precision of different raters and not to show the actual variability of the cylinders.

### 3.2. Human Total Arms

Table 4 shows the anthropometric characteristics of volunteers, separated by sex. Both men and women had a normal nutritional status, according to their mean BMI.

**Table 4:** Anthropometric variables: descriptive statistics.

Anthropometric Variable	Women (N = 15) Mean ± SD	Men (N = 15) Mean ± SD
Height (cm)	157.9 ± 7.2	171.2 ± 7.1
Weight (kg)	57.1 ± 12.5	69.6 ± 11.8
BMI (kg/m^2^)	22.8 ± 3.6	23.7 ± 3.3
Total Arm Length (cm)	34.2 ± 1.6	37.6 ± 1.9
Upper Arm Circumference (cm) ^a^	28.8 ± 4.2	30.9 ± 3.1

^a^ Evaluated at 60% of the distance between the acromion of the shoulder and the olecranon of the ulna.

#### 3.2.1. Accuracy

**Figure 4:**
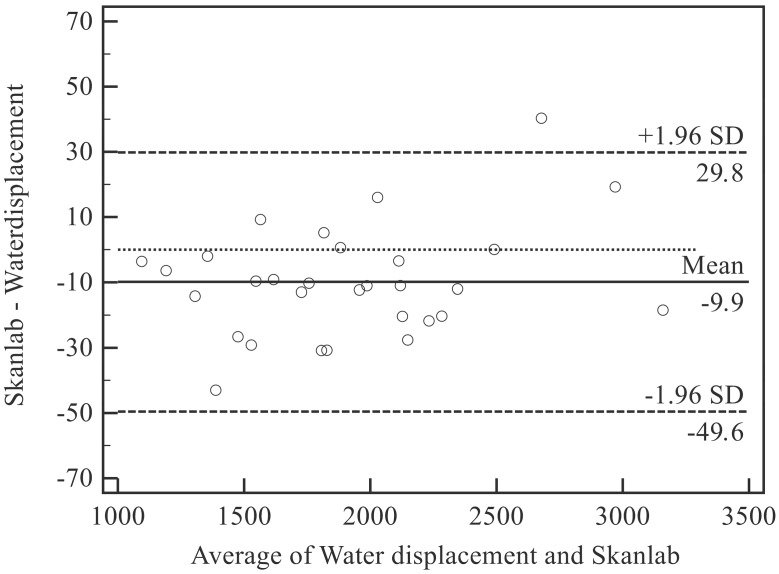
Bias and limits of agreement between SkanLab and Water displacement for human arms. SD, standard deviation.

The bias of SkanLab with respect to water displacement was equal to −9.9 mL (−0.6%) (LOA: −49.6 mL to 29.8 mL; −2.6% to 1.4%), showing a slight tendency towards underestimation (Figure 4; Table 5).

**Table 5:** Accuracy of SkanLab in measuring human arms (N = 30).

	Rater 1 Absolute Relative		Rater 2 Absolute Relative	
Bias ^a^	−13.6 mL	−0.8%	−6.1 mL	−0.4%
LOA ^b^	−60.1 to 32.8 mL	−3.3% to 1.7%	−54.4 to 42.2 mL	−2.8% to 2.0%

^a^ Mean difference between SkanLab and (water displacement); ^b^ limits of agreement.

The linear regression equation (*y* = −0.01*x* + 29.15; *R*^2^ = 0.086) showed no significant relationships between total arm volume (*x*) and accuracy (*y*, volumes measured by water displacement minus volumes measured by SkanLab).

On the contrary, the relationship between BMI (*x*) and accuracy (*y*, volumes measured by water displacement minus volumes measured by SkanLab) was highly significant (*y* = −2.55*x* + 69.19; *R*^2^ *=* 0.266; p = 0.004*)*.

#### 3.2.2. Reliability

As for inanimate objects, both SkanLab and water displacement showed high levels of intra- and inter-rater reliability (Table 6), with intra- and inter-rater ICC values falling within the limits of high reliability [37]. The intra- and inter-rater SEM of SkanLab and the water displacement technique was similar (Table 6).

**Table 6:** Reliability of water displacement and SkanLab in measuring human arms (N = 30).

	Water Displacement		SkanLab	
	Rater 1	Rater 2	Rater 1	Rater 2
	Mean ± SD (mL)	Mean ± SD (mL)	Mean ± SD (mL)	Mean ± SD (mL)
Replicate 1	1927.7 ± 491.9	1919.2 ± 489.9	1911.4 ± 495.4	1911.5 ± 501.8
Replicate 2	1922.2 ± 495.7	1920.2 ± 497.4	1911.3 ± 496.1	1915.7 ± 501.7
Intra-rater SEM ^a^	9.79	12.95	15.54	16.50
Intra-rater ICC ^b^	0.9996 (0.9991 to 0.9998)	0.9993 (0.9986 to 0.9997)	0.9990 (0.9978 to 0.9995)	0.9989 (0.9977 to 0.9995)
Inter-rater SEM ^a^	6.92		8.56	
Inter-rater ICC ^b^	0.9998 (0.9995 to 0.9999)		0.9997 (0.9994 to 0.9999)	

^a^ Standard error of measurement; ^b^ intraclass correlation coefficient.

#### 3.2.3. Duration of Measurement

The mean duration the volunteers were involved in the measurements for measuring volume was higher for the water displacement technique than for SkanLab, being respectively equal to 2′ and 3″ ± 29″ and 42″ ± 11″ (*p* ≈ 0.000).

The mean duration of data cleaning using MeshLab was equal to 8′ and 35″.

## 4. Discussion

Various methods can be used for determining arm volume: anthropometry, infrared technology [11], laser scanning and water displacement; the latter being considered the “gold standard” [38,39]. We have shown that SkanLab is a low-cost technique that is fast, reliable and accurate in measuring the volume of both inanimate objects and human arms when compared to the reference methods. In fact, the very low biases and narrow LOAs obtained in this study were similar or lower to the lowest values obtained with other methods (Table 7), while the intra- and inter-rater reliability was similar or higher and the measurement time shorter.

In particular, when measuring inanimate objects with respect to the geometrically-determined volume (Table 7), the accuracy of SkanLab, in terms of bias and LOA, was slightly worse than the very high one shown by laser scanning [16] and slightly better than that shown by Perometer [18]. However, Man *et al*. [18] do not mention the limits of the agreement nor show the raw data, hence reducing the informative value of the observed bias. The comparison with the water displacement technique is difficult because of the wide range of biases (from 2.7 mL, obtained in this research, to 120.7 mL). This variability is probably due to different rater expertise, instruments and experimental conditions. Moreover, the lack of information on LOA of some studies [18,30] does not allow a full interpretation of the results.

To our knowledge, there are no statistical indices on reliability, such as ICC or SEM, to be compared with the excellent values obtained in this study with inanimate objects. However, Mc Kinnon *et al.* [16] found high reliability evaluated by the coefficient of reproducibility (19.0 mL) using laser scanning.

When measuring human arms, SkanLab showed a similar or higher accuracy than that observed for circumferential methods, Perometer and laser scanning (Table 7). In fact, circumferential methods showed very high LOA and relatively low biases [8,9,13]. Perometer showed low biases [18,19] and a narrow LOA [19]. An alternative method based on the Kinect sensor showed a higher bias [27]. Laser scanning, despite its high-cost and complexity, showed a higher bias and a wider LOA [16]. On the basis of the high degree of concordance between perometry and DXA, Santìn and Ward [19] have proposed that these two methods could be used interchangeably.

SkanLab, as well as the other techniques, demonstrated very high levels of both intra- and inter-rater reliability, according to the Currier’s criteria [37]. In fact, ICC values for water displacement ranged between 0.94 [13] and 0.99 [8,9,40]; those for circumferential methods between 0.96 [13] and 0.99 [8,9,40,41]; those for Perometer [28,40] or Kinect [27] were equal or higher than 0.98. Furthermore, according to Mc Kinnon *et al.* [16], laser scanning showed a better reliability than water displacement, as measured by the coefficient of reproducibility (174 mL).

Lastly, the time needed for data acquisition with SkanLab (42″, in mean) was short and only slightly longer to that needed using Perometer (5″; [18]) and shorter than that of water displacement (10′, [18]; 2′, present study).

In comparison with the method recently proposed by Öhberg *et al*. [27], our procedure, based on a single mobile sensor instead of three fixed ones, appears more accurate, less expensive and easier to use. Its higher accuracy (−9.9 mL *vs*. 45.25 mL) is likely to be linked to the different methodological approach, which does not require calibration and uses all points of the three-dimensional mesh to compute the volume instead of limiting itself to 1 cm-wide limb segments. The higher usability and lower cost can be due to the simplified technical apparatus and to the adoption of free for non-commercial use and open source software.

While the arm dimension was not significantly related to accuracy, regression analysis showed a significant effect of BMI, as previously observed by Öhberg *et al.* [27]. This suggests that a better accuracy can be achieved with normal weight or slightly overweight people, while the total arms of obese ones could be overestimated.

In synthesis, SkanLab appears to be a promising technique for the measurement of total arm volume, combining similar accuracy and reliability of the reference methods, with the advantage of being faster, transportable, hygienic, completely safe and potentially low cost.

SkanLab is a good candidate for use in clinical routines, also being appropriate in patients with skin lesions or mobility impairment. In particular, it could be useful for measuring lymphedema. In fact, the differences within the limits of agreement observed with SkanLab would not be clinically important, considering that the diagnostic threshold for breast-cancer lymphedema is commonly based on a 200 mL (or 10%) volume difference between arms [42] and that an increase in arm volume between 5% and 10% has been suggested as the threshold for intervention to prevent lymphedema progression [43]. Given its high accuracy in terms of bias (0.6%) and LOA (−2.6% to 1.4%), SkanLab can also account for changes in the latent-stage lymphedema, hence being useful for prevention and monitoring of early interventions.

**Table 7:** Summary of literature results on accuracy.

Technique under Study	Standard	Bias ^a^ (mL)	LOA ^b^ (mL or %)	Sample Characteristics	Reference
Inanimate Objects (Cylinders)					
SkanLab	Geometry	−21.9	−62.0 to 18.2	Twelve cylinders (190 mL to 2002 mL)	Present Study
Water Displacement	Geometry	−2.7	−16.4 to 11.0		
		−7.6c	---	Eleven cylinders (10 mL to 4000 mL)	Lette *et al*., 2006 [30]
		−120.7	−348.1 to 106.7 ^c^	Seven cylinders (272 mL to 2042 mL)	Mc Kinnon *et al*., 2007 [16]
		52	---	A cylindrical object (1568 mL) measured 10 times	Man *et al*., 2004 [18]
Perometer	Geometry	34	---		
Laser Scanning	Geometry	−0.4	−14.7 to 13.9 ^c^	Seven cylinders (272 mL to 2042 mL)	Mc Kinnon *et al.*, 2007 [16]
Human Arms					
SkanLab	Water Displacement	−9.9	−49.6 to 29.8	Thirty healthy volunteers; right arm	Present Study
Circumferential Methods	Water Displacement	29.4 ^d^	−158.8 to 216.8	Forty-one breast cancer patients and 25 control subjects; right arm	Taylor *et al*., 2006 [13]
		75.4 ^e^	−110.2 to 260.2		
		52 ^f^	−282 to 386	Twenty-five breast cancer patients; surgical upper extremity	Megens *et al*., 2001 [8]
		40 ^g^	−194 to 274		
		---	479; 655 ^h^	Fifty patients with lymphedema; edematous arm	Sander *et al*., 2002 [9]
Kinect	Water Displacement	45.3	−36.3 to 126.8 ^i^	Twenty-five patients with lymphedema; both arms	Öhberg *et al*., 2014 [27]
Laser Scanning	Water Displacement	151.7	−227 to 53 ^l^	Ten volunteers; right arm	Mc Kinnon *et al*., 2007 [16]
Perometer	Water Displacement	74.1	---	Thirty-one healthy volunteers; dominant arm	Adriaenssens *et al*., 2013 [28]
Perometer	DXA^i^	0.7%	−7.7 to 6.3%	Measurements were performed on both whole arms	Santìn and Ward, 2014 [19]

^a^ Negative values represent underestimates with respect to the standard technique; ^b^ limits of agreement; ^c^ calculated from raw data; ^d^ anatomic landmarks; ^e^ distance from fingertips; ^f^ single truncated cone; ^g^ summed truncated cone; ^h^ values representing the range of different cumulative LOA, the lower plus higher limit value; ^i^ confidence interval; ^l^ dual energy X-ray absorptiometry.

A further application could be based on the combined use of total arm volume estimates and bioelectrical data. Some authors already consider bioelectrical-impedance spectroscopy (BIS), which is sensitive to the liquid volume of the upper extremity, and to extracellular water in particular, and appropriate for diagnosing changes in lymphatic volume [21]. An alternative approach could be based on the recently proposed vectorial bioimpedance analysis defined specific BIVA [44,45]. Specific BIVA, where bioelectrical values are corrected for body volume estimates, has been shown to produce accurate evaluations of the relative proportion of body fat and extracellular/intracellular water ratio, both aspects being related to lymphedema progression [46].

## 5. Conclusions

With respect to standard techniques, SkanLab proved to be a simpler, faster and safer procedure for assessing total arm volume, with very high levels of accuracy and reliability. The validated prototype can represent a basis for a low-cost instrument of wider use, suitable in various clinical applications. An automatization of the workflow through MeshLab scripts is planned in the future. Further validation is needed in clinical populations.
